# Clinical Evidence for the Use of Octenidine Dihydrochloride to Prevent Healthcare-Associated Infections and Decrease *Staphylococcus aureus* Carriage or Transmission—A Review

**DOI:** 10.3390/pathogens12040612

**Published:** 2023-04-18

**Authors:** Robin Köck, Luisa Denkel, Andrea T. Feßler, Rudolf Eicker, Alexander Mellmann, Stefan Schwarz, Christine Geffers, Nils-Olaf Hübner, Rasmus Leistner

**Affiliations:** 1 Institute of Hygiene, University Hospital Münster, 48149 Münster, Germany; 2 Hygiene and Environmental Medicine, University Hospital Essen, 45147 Essen, Germany; 3 Institute of Hygiene and Environmental Medicine, Charité—Universitätsmedizin Berlin, 12203 Berlin, Germany; 4 Institute of Microbiology and Epizoonotics, Freie Universität Berlin, 14163 Berlin, Germany; 5 Veterinary Centre for Resistance Research (TZR), Freie Universität Berlin, 14163 Berlin, Germany; 6 Institute for Hygiene and Environmental Medicine, University Medicine Greifswald, 17489 Greifswald, Germany; 7 Division Gastroenterology, Infectious Diseases and Rheumatology, Medical Department, Charité Universitätsmedizin Berlin, 12200 Berlin, Germany

**Keywords:** wound infection, postoperative, blood cultures, nosocomial, MRSA, decontamination, eradication, CLABSI

## Abstract

Background: The antiseptic agent octenidine dihydrochloride (OCT) is used for skin preparation, for *Staphylococcus aureus* decolonization, and within bundles for the prevention of catheter-related or surgical site infections (SSIs). Here, we review the evidence for the effects of OCT from clinical studies. Methods: Review of studies published in the Medline, Scopus, and Cochrane databases until August 2022, performed in clinical settings and reporting on effects of OCT on *S. aureus* carriage/transmission, SSI prevention, and prevention of intensive care unit (ICU)-related or catheter-related bloodstream and insertion site infections. Results: We included 31 articles. The success of *S. aureus* decolonization with OCT-containing therapies ranged between 6 and 87%. Single studies demonstrated that OCT application led to a reduction in *S. aureus* infections, acquisition, and carriage. No study compared OCT for skin preparation before surgical interventions to other antiseptics. Weak evidence for the use of OCT for pre-operative washing was found in orthopedic and cardiac surgery, if combined with other topical measures. Mostly, studies did not demonstrate that daily OCT bathing reduced ICU-/catheter-related bloodstream infections with one exception. Conclusions: There is a need to perform studies assessing the clinical use of OCT compared with other antiseptics with respect to its effectiveness to prevent nosocomial infections.

## 1. Introduction

Antiseptics are used to reduce the microbial load on a patient’s skin, mucosa, or wounds to prevent endogenous healthcare-associated infections (HAIs). Octenidine dihydrochloride (OCT) is a bispyridine-type antiseptic with activity against gram-positive and gram-negative bacteria.

The killing mechanism of OCT on gram-negative bacteria is proposed to be unspecific. After binding to the bacterial surface, it penetrates through the lipopolysaccharide layer and interacts with fatty acyl chain regions of the outer membrane, causing a lipid disorder, which finally leads to bacterial cell lysis [1]. Similarly, inducing membrane disorders is also observed in gram-positive bacteria [2].

Its cationic properties allow OCT to bind to negatively charged surfaces including the envelopes and membranes of microbial cells as well as human cells [3]. It is assumed that this facilitates the ability to form a long-term persistent residue that has a biocidal effect against microorganisms (re-)emerging, e.g., from sebaceous glands or hair follicles after disinfection [4]. Another advantage is that some experiments have demonstrated that OCT has an effect even on microorganisms that are sheltered within biofilms [4]. Its allergenic potential seems to be low. Moreover, long-term experience with the substance, which has been marketed more than forty years ago, has suggested a rather low rate of resistance development [4,5,6], although some authors observed a rapid increase in minimum inhibitory concentrations after OCT introduction [7].

However, some clinicians are reluctant to use OCT-containing preparations for peri-operative antisepsis or for catheter insertion because they are colorless. They perceive that this might increase the likeliness that relevant areas are inadvertently spared when applying the disinfectant or OCT could be accidentally mistaken for substances used for intravenous injection. In addition, OCT may cause tissue necrosis when applied in deep-seated, undrained wounds similar to a non-excretable foreign body [8,9].

Microbiological studies have demonstrated that OCT decreases the load of bacteria that colonize or contaminate the skin, the mucosal membranes, or saliva [4,5]. However, the results of such microbiological studies do not necessarily reflect the clinical effectiveness of OCT to prevent HAIs, because, for example, the mentioned disadvantages of the colorless substance might result in application failure. For other antiseptics, such as chlorhexidine, their effectiveness has been evaluated and compared with alternative substances for various clinical outcomes like ventilator-associated pneumonia [10], skin preparation before caesarean section [11], intensive care unit (ICU)-acquired (e.g., central-line associated) bloodstream infections (BSIs) [12,13] or hospital-acquired infections [14,15], nasal *Staphylococcus aureus* decontamination [16], or surgical site infections (SSIs) [17].

Therefore, the aim of this review is to summarize the evidence for the effectiveness of OCT in clinical settings and answer three major research questions:

- What is the impact of using OCT-containing solutions for the decolonization of known carriers of *S. aureus* including methicillin-resistant *S. aureus* (MRSA) and methicillin-susceptible *S. aureus* (MSSA) on transmission or infection?
- What is the impact of using OCT-containing solutions directly before surgical interventions (pre-incisional antisepsis or skin preparation) or universally and multiple times before and after surgical interventions (pre-operative bathing/washing) on the occurrence of SSIs?
- What is the impact of using OCT-containing solutions directly before catheter insertion (pre-insertional antisepsis) or universally and multiple times among patients at risk on the occurrence of ICU- or catheter-related bloodstream infections and infections at catheter insertion sites?

Answering these questions shall allow for conclusions about the current state of clinical evidence regarding OCT application and shall elucidate fields where clinical investigations are needed.

## 2. Materials and Methods

This review was performed considering the PRISMA guidelines [18]. We searched Medline, Scopus, and the Cochrane databases for the term “octenidine” without language or time restrictions (until 21 August 2022). Titles and abstracts of the identified articles were screened by assessing whether they met the entry criteria. A detailed description of the entry and exclusion criteria, as well as the process of data extraction, is shown in Table S1. The primary outcomes assessed in this review were (i) the efficacy of *S. aureus* decolonization or the incidence of *S. aureus* infections after treatment with OCT (alone or in combination with other substances), (ii) the incidence of SSIs (including all types of superficial and deep-seated SSIs), and (iii) the incidence of ICU- or catheter-related bloodstream infections and catheter insertion site infections. Secondary outcomes were the rate of nosocomial *S. aureus* transmission and effects of OCT (on decolonization, transmission and so on) when applied intranasally.

## 3. Results

### 3.1. Literature Search Results

**Figure 1:**
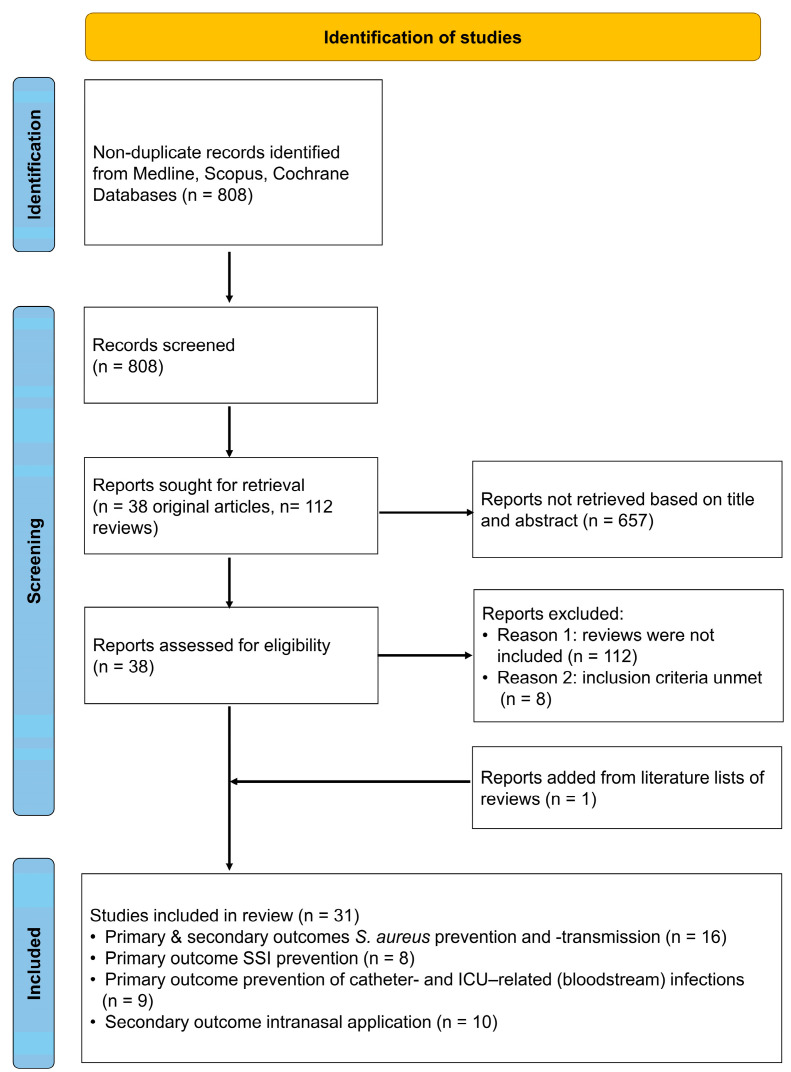
Search flow diagram. SSI = surgical site infection, ICU = intensive care unit, *S. aureus* = *Staphylococcus aureus*.

Overall, the literature search identified 808 non-duplicate articles (Figure 1). After evaluation, 31 studies were included in this review (Table 1) [19,20,21,22,23,24,25,26,27,28,29,30,31,32,33,34,35,36,37,38,39,40,41,42,43,44,45,46,47,48,49].

Of these studies, 16 were (at least partly) conducted in Germany (52%), while the others were performed in Switzerland and Singapore (both *n* = 4), Austria and the United Kingdom (both *n* = 3), and Lithuania and Turkey (both *n* = 1). Most investigations were single-centre studies (27/31, 87%). Twelve of the 31 studies (39%) were supported by manufacturers of the antiseptic products evaluated in the respective study.

As shown in Table 1, 16 of the included studies assessed the preventive effects on *S. aureus*/MRSA spread, carriage, and infection (entry criterion 1); six studies assessed the effects on SSIs (entry criterion 2); and nine on catheter-related infections (entry criterion 3).

The study outcomes, designs, and detailed results are shown in Table S2 (Supplementary Material). An overview of the OCT-containing products used in the study is given in Table S3 (for other than nasal application sites) and Table S4 (for nasal application). Briefly, the effects for the three study questions as well as primary and secondary outcomes assessed in this review were as follows.

### 3.2. S. aureus Decolonization, Infection Prevention, and Nosocomial Transmission

Among 16 studies, 9 were noncomparative, i.e., they used OCT among persons identified as *S. aureus* carriers and described effects on carriage rates or the success of decolonization without comparing OCT to the effects of other antiseptics or antibiotics [21,24,25,27,28,29,30,31,32]. The interventions were too heterogeneous to compare the decolonization success rates of these studies because of the variation in the treatment regimens, the microbiological techniques to control the success of the therapy, and the time intervals in which patients were followed up after the end of the treatment. The highest success rate (87% decolonization) was reported by a study that used OCT with only few vaginal carriers included and that combined topical treatment with systemic antibiotics [21]. The lowest success rate (6%) was found in a retrospective assessment of the efficacy of MRSA decolonization treatments where OCT body washes were planned for all MRSA-positive patients, but application of mupirocin nasal ointment only for those in whom nasal carriage was detected [25]. However, in this study, many data were missing and the authors did not record to what extent the planned interventions were carried out [25].

Of the remaining seven studies that compared the effects of OCT use to either other antiseptics or no antiseptic use, three evaluated the effects of nasal OCT (the results for this secondary outcome are summarized below) [19,20,22]. In the other four studies, one reported no reduction in nosocomial MRSA acquisitions (*p* = 0.31) and MRSA clinical infections (*p* = 0.96) in a setting where patients received OCT body washes pending the results of an MRSA admission screening (baseline phase). Those patients identified as MRSA carriers in the screening were additionally treated with OCT body washes for at least seven days [26]. In contrast, Spencer et al. found that washing patients not colonized with MRSA (as determined by screening) with water and soap versus OCT for five days led to a reduction (*p* < 0.01) in MRSA acquisition rates (assessed by weekly screenings) [33]. On a neonatal ward, introduction of an MSSA screening and OCT washing of all infants, as well as mupirocin treatment of all those infants colonized with MSSA, resulted in a reduction in the incidence rate of MSSA-attributable infections (*p* = 0.024) [34].

We identified only one study that assessed the effects of OCT versus chlorhexidine bathing on MRSA carriage rates; that is, Chow et al. found that, compared with no antiseptic bathing, bathing of all patients with either OCT (combined with nasal OCT for known MRSA carriers) reduced the prevalence of MRSA carriage assessed in point prevalence surveys. A similar reduction was found for chlorhexidine bathing [23].

### 3.3. Incidence of Surgical Site Infections

We identified two studies that assessed the effects of OCT used as a pre-incisional antiseptic in the operating theatre on the occurrence of SSIs [37,39]. In one study, the eyes of patients undergoing blepharoplasties were treated with an OCT-based product using a defined technique. Overall, 0/352 patients developed SSIs. However, this study had no control group, where antisepsis was performed with other substances or in which a baseline rate of SSIs was assessed [39]. In another study, a 50% reduction in SSIs was observed among patients receiving arterial reconstructive surgery after the implementation of a plethora of interventions, one of which was changing from an alcohol-based pre-operative disinfection to an OCT-based product [37]. However, as other factors, such as pre-operative antiseptic bathing and nasal gel, as well as surgical techniques, among others, were changed simultaneously, the effects attributable to that specific component of the intervention were not evaluated [37].

Four studies assessed the effects of OCT use when patients undergoing surgical interventions performed whole-body washes with OCT-based products prior to surgery [35,36,38,40] in combination with mupirocin nasal ointment [35,36], chlorhexidine washes for non-bedridden patients [38], and nasal OCT [40].

One retrospective study among patients receiving breast implant surgery found no difference between the control group without antiseptic washing versus the intervention group with OCT-based washing regarding the occurrence of minor SSIs (8/101 vs. 10/96, *p* = 0.543) and major SSIs (2/101 vs. 3/96, *p* = 0.584) [35].

A before-and-after study among cardiac surgical patients revealed that the overall SSI rate was not significantly changed between a control phase without universal washing with either OCT or chlorhexidine-based products (8.6%, 81/945) versus the intervention phase (6.9%, 58/842) (*p* = 0.19) [38]. However, in multivariable analysis, the intervention was associated with an odds ratio of 0.61 (95% confidence interval, 0.41–0.91; *p* = 0.015) regarding the occurrence of any SSI. This effect was only due to a decrease in superficial SSIs (10/646 vs. 8/196, *p* = 0.032; risk reduction 0.29 (0.15–0.58), *p* < 0.001). A major limitation of this study (with respect to the questions of this review) was that body washes in the intervention group were carried out using two different antiseptics, i.e., chlorhexidine for non-bedridden patients and OCT only for bedridden patients. In the results, these two groups are not stratified, and it remained unspecified how many patients were actually treated with each of the two antiseptics [38].

Another before-and-after study also observed no significant effects on overall SSI rates among patients undergoing cardiac surgery [40] when comparing a control (without antiseptic washing) and an intervention group where OCT-based body washes were applied (15.4% vs. 13.3%, *p* = 0.39). Differences were observed for SSIs at the venal harvest site (2.5% vs. 0.5%, *p* = 0.01) and, in patients with median sternotomy, for organ/space sternal SSIs (1.9% vs. 0.3%, *p* = 0.04). However, there was a trend towards more deep incisional sternal SSIs in the intervention group (1.2% vs. 2.9%, *p* = 0.08) and, in multivariate analysis, no significant protective effect of the intervention was found (odds ratio 0.79, 95% confidence interval 0.53–1.15, *p* = 0.27) [40].

In a retrospective cohort study, patients undergoing elective prosthetic joint surgery (mostly hip replacements) were treated with OCT washing for five days prior to surgery [36]. Those patients identified as MSSA carriers were additionally treated with mupirocin nasal ointment. This intervention led to a reduction in the overall rate of prosthetic joint infections (*p* = 0.03) and the rate of prosthetic joint infections specifically due to MSSA (*p* < 0.0001).

### 3.4. Incidence of ICU- or Catheter-Related Bloodstream Infections and Insertion Site Infections

Nine studies assessed the effects of OCT on ICU- or catheter-related infections, including three randomized trials.

Among these, four studies assed the effects of daily whole-body washes among risk patients with central lines. One large cluster-randomized, controlled, multi-center trial [43] compared routine care for patients on ICUs (without antiseptic body washes) to daily whole-body washes with either OCT or chlorhexidine and assessed the incidence density of central-line-associated bloodstream infections (CLABSIs). This study found no significant differences of both OCT and chlorhexidine body washes compared with the control group (adjusted incidence rate ratios of CLABSI were 0.69 (95% CI 0.37–1.22, *p* = 0.28) in the chlorhexidine group and 1.22 (95% CI 0.54–2.75, *p* = 0.65) in the OCT group compared with routine care). However, the authors concluded that the trial had a high likelihood of being underpowered because CLABSI rates in the routine care group were much lower than assumed [43].

Moreover, we found three non-randomized interventions regarding the use of OCT-based whole-body washes: One intervention introduced universal nasal treatment with OCT for five days after ICU admission and daily OCT washing during the whole ICU stay [46]. The authors observed a significant reduction in ICU-acquired bloodstream infections in medical (incidence rate ratio 0.78; 95% CI 0.65–0.94), but not in surgical ICUs [46]. Another study assessed the effects of daily OCT whole-body washes on the incidence of CLABSIs, which decreased from 2.03/1000 central venous catheter-days in a baseline period without antiseptic washing to 0.82/1000 in the washing phase (incidence rate ratio 0.40, 95% CI 0.06–1.71, *p* = 0.254) [41]. Similarly, the third study newly introduced daily OCT-based whole-body washes for all patients in an ICU and observed no significant effects on the occurrence of bloodstream infections (2.98/1000 patient-days before intervention vs. 2.06/1000 patient-days post intervention, *p* = 0.147) [47].

Five other studies assessed the effects of OCT when applied before the insertion of catheters and/or thereafter when changing dresses. Among these, in one trial, patients with non-tunneled central lines were randomized to receive antiseptic insertion site treatment with OCT/alcohol versus alcohol alone [44]. This study found less CLABSIs in the OCT group (8/194 vs. 16/193), but no significant difference (*p* = 0.081), although quantitative microbiological assessments revealed that the degrees of bacterial contamination at the insertion site and at catheter tips were significantly reduced in the OCT group. Another randomized trial used OCT for pre-insertional antiseptics of central venous catheters in one ICU, as well as for insertion site care thereafter [42]. When randomizing patients into three groups (consisting of 19 patients each) where antiseptic treatment was performed with chlorhexidine versus povidone iodine versus OCT, catheter-related sepsis occurred in 0% versus 10.5% versus 20.5% (*p* < 0.001) of the patients and catheter insertion site colonization in 0% versus 26.3% versus 21.5% (*p* < 0.001), thus indicating the disadvantages of OCT-based disinfection. However, besides the very small number of patients included in this study, many details of the study design remained unspecified (e.g., absolute numbers of infection, definition of infection, length of treatment, and infections per catheter-days) or controversial (technique of non-quantitative insertion site culture), which makes it difficult to draw robust conclusions [42].

Another study evaluated whether implementing a new care bundle for long-term central venous access devices (CVADs) including OCT-based disinfection of catheter hubs influenced the occurrence of bloodstream infections [45]. This study found no significant reduction associated with the intervention. When the occurrence of catheter infections of thoracic epidural catheters was assessed among patients receiving elective major abdominal surgery [49], no significant difference was found between patients where disinfection was carried out with an alcoholic product alone versus disinfection with OCT/alcohol (6/1120 vs. 10/1635, *p*= 0.797). Finally, a noncomparative study observed an incidence density of catheter infections of 2.39 per 1000 catheter-days when using povidone–iodine as an antiseptic for insertion of non-tunneled central venous catheters and OCT thereafter when dresses were changed [48].

### 3.5. Secondary Outcome—Nasal Application of OCT

Intranasal application of OCT was performed in ten of the included studies measuring different primary outcomes [19,20,22,23,24,28,30,40,41,46]. How often the nasal gel was applied daily was unspecified in five studies, while it was used thrice daily in three, twice daily in one, and once daily in one study (Table S4). The concentration of the gel was not mentioned in eight of the studies (Table S4).

However, six of the ten studies used intranasal OCT within preventive bundles, but did not assess the effects of nasal OCT compared with other antiseptics or antibiotics, or the effects attributable to nasal OCT [23,24,28,40,41,46].

The only comparative evaluation of OCT nasal gel versus other nasally applied substances demonstrated that OCT nasal gel was inferior to reduce *S. aureus* carriage among patients receiving elective hip and knee joint arthroplasty compared with mupirocin nasal ointment or neomycin [19]. Of all *S. aureus* carriers, 35/322 (10.9%) of those treated with mupirocin (2×/d), but 415/830 (50%) of those treated with OCT (2×/d) and 57/624 (9.1%) of those treated with neomycin/chlorhexidine (4×/d), still carried *S. aureus* after a treatment course of five days (*p* < 0.0001) [19]. A limitation of this study might be that the application intervals of the different substances varied between twice and four times daily.

Similar results were found in a small observational study in which eight of eight persons [30], in whom two decolonization attempts using OCT nasal gel (in combination with OCT-based body washes and mouth irrigation) had failed, were successfully treated with mupirocin nasal ointment (in combination with the same OCT-based products for body washes and mouth rinse). However, besides the nasal ointment, a professional dental cleaning, as well as targeted screenings and treatments of the household members of the eight persons, were also offered in parallel to the mupirocin-based decolonization attempt [30].

Aung et al. routinely performed OCT whole-body washes for all patients in a dermatology ward [20]. After starting to additionally treat MRSA carriers among these patients with OCT nasal gel for five days, the nosocomial MRSA acquisition rate in the ward decreased (*p* = 0.046) and the number of MRSA carriers who were screened negative at discharge increased (from 6/92 (9.7%) in the baseline period to 10/47 (21.3%) in the nasal treatment period) [20].

In addition, in a ward where universal chlorhexidine baths were used for all patients, the MRSA acquisition rate per 1000 patient-days decreased (7.0 vs. 4.4/1000 patient-days, *p* < 0.001) after adding nasal OCT for those patients identified as MRSA carriers [22].

## 4. Discussion

In this review, we aimed to assess the clinical evidence for the effects of OCT. Most studies included in this review are from European countries, and Germany (16/31) in particular. One of the reasons for this geographic predominance might be that chlorhexidine, which is often used in other countries, is marketed in Germany only within special, single-use applicators (containing 1–20 mL of the antiseptic) and not, like other antiseptics, in packages of 500–2000 mL. This might be perceived as being more expensive, less feasible for individual dosing, and causing plastic waste. Hence, OCT might be considered as an alternative by many clinicians and infection control specialists. As summarized recently, the in vitro effects of OCT are well evaluated [5]. However, this review aimed to focus on preventive effects demonstrated in application studies and assessing clinical outcomes, as these are most important for decision-making in the clinical setting. Studies on chlorhexidine-based antiseptics found differences in the effectiveness to reduce HAIs depending on the concentration [50]. In this context, it is a clear limitation of the majority of studies included in this review that either the OCT-containing product or the concentration of OCT within the respective product were not specified (Tables S3 and S4). Moreover, the heterogeneity of surveillance techniques used in the studies and diversity of outcomes measured (e.g., ICU-acquired bloodstream infections vs. catheter-related bloodstream infections vs. all infections) prevented performing a meta-analysis.

### 4.1. Outcomes of S. aureus Decolonization and Prevention of S. aureus Infection and Transmission

The majority of identified studies from clinical settings investigated OCT within preventive bundles to decolonize patients or healthcare workers from *S. aureus* (mostly MRSA) carriage in order to contain the spread of *S. aureus* in wards and, simultaneously, to prevent healthcare-associated infections. In general, the principle of “search and destroy” or “screening and decolonization” is well evaluated as being effective [51]. However, the success rates of OCT-based *S. aureus* decolonization therapies described in noncomparative studies varied significantly (6% vs. 87% success). As in vitro *S. aureus* strains prevalent in Germany were found to be susceptible to OCT [52], this might mainly be because of divergent decolonization protocols (e.g., with or without standard nasal irrigation, treatment of pharyngeal carriage, and systemic antibiotics). A major factor might also be different follow-up protocols to control the success of the decolonization therapy (e.g., a different number of swabs from different body sites). Hence, these results are barely comparable and can mainly be used to conclude that OCT-based decolonization has an (unquantified) clinical in vivo effect.

However, we identified two investigations assessing the effects of OCT washing on MRSA-acquisition rates. While applying OCT body washes versus washing with water and soap among patients not colonized with MRSA led to a decrease in nosocomial MRSA acquisition in a ward [33], washing patients with OCT, pending the results of an MRSA admission screening as well as MRSA identified as carriers for seven days thereafter, did not result in a reduction in MRSA acquisitions (*p* = 0.31) or MRSA clinical infections (*p* = 0.96) [26]. In addition, one study showed that OCT washing of all patients in a neonatal ward, and additionally treating MSSA carriers among these patients with mupirocin, resulted in a decrease in MSSA infections [34]. Moreover, we identified one study comparing the effects of routine OCT versus chlorhexidine bathing, which demonstrated that both antiseptics were equally effective to reduce the prevalence of MRSA carriage [23].

Hence, these investigations might lead to the conclusion that there is weak evidence that routine OCT bathing (at least as a component of preventive bundles) of patients who are not known *S. aureus* carriers can reduce MSSA/MRSA spread in a clinical setting.

### 4.2. Outcome Prevention of Surgical Site Infections

Another question raised in this review was whether there are studies that assessed the clinical effects of OCT to prevent SSIs or that used OCT as a pre-incisional antiseptic. Except one noncomparative study among patients undergoing eye surgery [39], we found only one small study where SSI rates were monitored before and after changing the pre-incisional skin preparation from alcohol to OCT [37]. However, in the latter study, this change was incorporated as one component of a bundle of interventions, and it was not possible to assess whether the observed reduction in SSIs was attributable to the introduction of OCT [37]. In consequence, evidence to support the clinical use of OCT as a pre-incisional antiseptic is very rare. We conclude that the arguments of clinicians who fear an application failure of the colorless substance OCT, which could corroborate its in vitro effects, are justified and have not yet been adequately addressed in clinical studies.

Apart from that, we identified four studies in which OCT-based washing was performed before elective surgical interventions. In breast surgery, no preventive advantages were observed [35]. In contrast, in cardiac surgery [38,40], one study demonstrated that antiseptic washing with either chlorhexidine or OCT reduced superficial SSIs, while overall SSI rates and rates for deep-seated SSIs remained stable [38]. A major limitation of that study was that it remained unclear how many of the patients were treated with chlorhexidine and how many were treated with OCT (which was only used for bedridden persons) [38]. The second study among cardiac patients, although observing a reduction in SSIs at venal harvest and median sternotomy sites, found that deep incisional sternal SSIs were even more frequent in the OCT group (1.2% vs. 2.9%, *p* = 0.08) and, in multivariate analysis, no significant protective effect of the intervention was found (odds ratio 0.79, 95% confidence interval 0.53–1.15, *p* = 0.27) [40]. In orthopedic surgery, one study demonstrated that combining OCT bathing before elective prosthetic joint surgery with nasal mupirocin treatment of *S. aureus* carriers after screening resulted in decreasing overall SSI rates and, specifically, MSSA infections [36].

Hence, the role of OCT bathing prior to surgical interventions remains controversial. If implementing this intervention is considered, e.g., if local infection surveillance indicates high SSI rates, it should be considered to implement nasal application of mupirocin to prevent *S. aureus* infection in parallel.

### 4.3. Outcome Prevention of ICU-/Catheter-Related Bloodstream Infections and Insertion Site Infections

The intervention of chlorhexidine-based washing of patients on ICUs has been evaluated in many studies, with uncertain results overall [14]. Among the studies identified in this review, four evaluated whether effects can be demonstrated using OCT for whole-body washes. In a large cluster-randomized trial [43], daily OCT washes did not result in a reduction in CLABSIs compared with routine washing with soap and water. However, in that trial, chlorhexidine also failed to demonstrate this effect. Comparing the effects of chlorhexidine versus OCT showed a tendency that chlorhexidine was more successful (adjusted incidence rate ratios of CLABSIs were 0.69 (95% CI 0.37–1.22, *p* = 0.28) in the chlorhexidine group and 1.22 (95% CI 0.54–2.75, *p* = 0.65) in the OCT group compared with routine care), but the authors did not calculate whether infection rates in the two groups differed significantly [43]. This information was added in a post-hoc analysis of this randomized trial. The authors reported that, in the chlorhexidine group, the incidence density of CLABSIs was reduced compared with a baseline period without antiseptic washing (1.48 vs. 0.90 CLABSIs per 1000 central-line days, *p* = 0.0085), while no reduction was observed in the OCT group (1.26 vs. 1.47 CLABSIs per 1000 central-line days, *p* = 0.8735) [53]. The preventive effect of chlorhexidine was particularly found in ICUs with ≥0.8 CLABSIs per 1000 central-line days at baseline [53].

Three non-randomized investigations observed divergent results. When patients in ICUs were treated with nasal OCT and OCT washing, ICU-acquired bloodstream infections were reduced in medical, but not in surgical ICUs (incidence rate ratio 0.78; 95% CI 0.65–0.94) [46]. Similarly, two further studies observed tendencies towards decreasing infection rates, but no significant effects associated with the introduction of OCT-based washing [41,47].

In summary, no study clearly demonstrated until now that using OCT-based whole-body washes in ICUs led to a reduction in CLABSIs or ICU-acquired bloodstream infections; only one investigation found significant effects when additionally applying OCT nasal gel [46]. Recently, a large German multi-center study assessed OCT whole-body washes versus washing with a placebo on ICUs [54]. The preliminary results from >90,000 patients indicated a reduction in ICU-acquired blood cultures, positive with microorganisms that were not simultaneously detected in other microbiological specimens [55]. However, it is a major limitation of this study that it did not assess whether these bacteremia cases were cases of primary bacteremia according to NHSN definitions [56] or whether they were associated with suspected infections at other sites or occurred among patients with central lines.

Besides daily whole-body washes, OCT might also be used for antiseptic treatment of catheter insertion sites prior to insertion and thereafter when changing the dresses. We found four studies evaluating whether this improved infection rates compared with other antiseptic regimens, none of which demonstrated a significantly favorable effect of OCT [42,44,45,49]. This shall not obscure that data indicate a reduction in bacterial contamination (in terms of bacterial loads counted in colony forming units) of catheter insertion sites when OCT is applied in addition to other antiseptics [44,57].

### 4.4. Secondary Outcome Intranasal OCT Use

For the purpose of nasal *S. aureus* decolonization, mupirocin 2% nasal ointment is a standard therapy, which has been demonstrated to be effective in numerous studies [51]. Measuring the success rates of mupirocin-based therapies highly depends on the duration of follow-up cultures taken to confirm or exclude the re-emergence of carriage, but ranges between 90% (one after treatment) and 60% (after longer follow-up periods) [58]. It has often been argued that preventive mupirocin use could facilitate the occurrence of resistance. Indeed, studies have shown that mupirocin resistance emerged in patient populations that were frequently treated [59]. Against this background, OCT nasal gel could be an alternative for decolonizing *S. aureus* carriage in the nares. While several studies identified in this review used OCT nasal gel as a part of their interventions [19,20,22,24,28,30,41,46], we identified only one prospective study that directly compared the effectiveness of mupirocin, OCT, and chlorhexidine/neomycin nasal ointments for eradicating *S. aureus* [19]. This study clearly showed that OCT 0.1% was the least effective agent. Hence, if targeted and safest decolonization of *S. aureus* is the aim of the intervention, and mupirocin resistance rates are low, nasal OCT 0.1% should be discouraged. However, Aung et al. and Chow et al. have demonstrated that, compared with no nasal treatment, MRSA transmission decreased and decolonization was more successful when nasal OCT was applied for MRSA carriers [20,22].

## 5. Conclusions

In this review, we found that there is rarely evidence that quantifies the clinical effects of OCT on *S. aureus* carriage rates. For treating nasal carriage, the effects were proven, but it was demonstrated that, compared with other topical agents, nasal OCT was less effective. Studies comparing the effectiveness of OCT versus other antiseptics for skin preparation and for daily whole-body washing to prevent SSIs and catheter-related bloodstream infections were rare. Hence, concerns of clinicians regarding the use of the substance cannot be completely alleviated with evidence from clinical studies. Overall, we conclude that investigations comparing the effects of OCT versus alternative substances on defined clinical outcomes are needed.

## Figures and Tables

**Table 1:** Overview of studies assessing the effects of octenidine dihydrochloride (OCT).

Study (Ref.)	Year	Country	Design ^1^	Setting ^2^	Commercial Support ^3^
Studies using OCT in bundles to prevent *S. aureus* or MRSA spread and infection among patients with confirmed carriage					
Allport [19]	2010–2018	UK	Retrospective cohort	1 hospital, orthopedics	ND
Aung [20]	2016–1018	Singapore	Controlled B/A	1 hospital, dermatology	ND
Buehlmann [21]	2002–2007	Switzerland	Noncomparative	1 UH	ND
Chow [22]	2013–2019	Singapore	Interrupted time-series	1 RH	Schülke/Mayr
Chow [23]	2014–2016	Singapore	Controlled B/A	1 RH, 2 CH	Schülke/Mayr
Danilevicius [24]	2011–2012	Lithuania	Noncomparative	1 UH	Schülke/Mayr
Hansen [25]	1999–2004	Germany	Noncomparative	1 TH, medical wards	ND
Harris [26]	2011–2013	Singapore	Cluster crossover	1 TH	Schülke/Mayr
Kaminski [27]	2001–2002	Germany	Noncomparative	1 TH	ND
Pichler [28]	2016	Austria	Noncomparative	1 TH	ND
Rengelshausen [29]	unknown	Germany	Noncomparative	1 hospital, hemodialysis	ND
Richter [30]	2016	Germany	Noncomparative	1 UH, 1 neonatal ICU	ND
Rohr [31]	1998–2002	Germany	Noncomparative	2 hospitals	Schülke/Mayr
Sloot [32]	1997–1998	Germany	Noncomparative	1 hospital	Schülke/Mayr ^4^
Spencer [33]	2009–2011	UK	Retrospective cohort	1 hospital, 1 ICU	ND
Wisgrill [34]	2011–2016	Austria	Retrospective cohort	1 UH, neonatal wards	ND
Studies using OCT for pre-incisional antisepsis or universally among patients and assessing the effects on surgical site infections					
Hachenberg [35]	2016–2019	Germany	Retrospective cohort	1 municipal hospital	ND
Jeans [36]	2007–2014	UK	Retrospective cohort	3 hospitals, orthopedics	ND
Karl [37]	2015–2017	Germany	Prospective cohort	1 TH, vascular surgery	ND
Kohler [38]	2009–2010	Switzerland	Controlled B/A	1 TH	ND
Matiasek [39]	2010–2016	Austria	Noncomparative	1 UH, plastic surgery	ND
Reiser [40]	2013–2014	Germany	Controlled B/A	1 UH	Schülke/Mayr
Studies using OCT universally among defined patients and assessing the effects on ICU-/catheter-related bloodstream and insertion site infections					
Baier [41]	2012–2017	Germany	Retrospective cohort	1 UH, 1 ICU	ND
Bilir [42]	unknown	Turkey	Randomized trial	1 UH, 1 ICU	ND
Denkel [43]	2017–2018	Germany	Cluster-randomized controlled trial	68 hospitals, 72 ICUs	Sage/Stryker, Schülke/Mayr
Dettenkofer [44]	2002–2005	Switzerland/Germany	Double-blind randomized controlled trial	2 UH, haematology/surgical unit	Schülke/Mayr
Furtwängler [45]	2009–2013	Germany	Observational study	1 UH, paediatric cancer centre	Becton Dickinson
Gastmeier [46]	2013–2015	Germany	Interrupted time-series	1 UH, 17 adult ICUs	ND
Messler [47]	2012–2014	Germany	Before-and-after	1 UH, 1 surgical ICU	Schülke/Mayr
Tietz [48]	2000–2001	Switzerland	Noncomparative	1 UC, bone marrow transplant unit	Schülke/Mayr
Vogelsang [49]	2010–2018	Germany	Retrospective cohort	1 UC, anaesthesiology	ND

^1^ B/A = before-and-after. ^2^ ICU = intensive care unit. TH = tertiary care hospital. UH = university hospital, RH = rehabilitation hospital, CH = community hospital. ^3^ Names of companies distributing antiseptics that supported the study. ND = not declared. ^4^ co-authorship.

## Data Availability

Not applicable.

## Supplementary Materials

The following supporting information can be downloaded at https://www.mdpi.com/article/10.3390/pathogens12040612/s1. Table S1: Detailed description of the entry criteria, exclusion criteria and data extraction process, Table S2: Data extraction table, Table S3: Overview of octenidine-containing products used for non-nasal application sites, Table S4: Overview of studies in which octenidine-containing products were used for nasal application.
