# Supplementary material for: Clinical Evidence for the Use of Octenidine Dihydrochloride to Prevent Healthcare-Associated Infections and Decrease Staphylococcus aureus Carriage or Transmission—A Review
Source: Pathogens. 2023 Apr 18;12(4):612. doi: 10.3390/pathogens12040612 (PMC10145019; doi:10.3390/pathogens12040612)
Supplement: Supplementary file 1 [file pathogens-12-00612-s001.zip › pathogens-2261955-supplementary.pdf]

pathogens

MDPI

Supplementary Material

Table S1. Detailed desription of the entry criteria, exclusion criteria and data extraction process.

Titles and abstracts of the identified articles were screened by the authors RK by assessing whether they met the entry criteria.

Entry criteria

We included i.) articles assessing effects of OCT on *S. aureus* carriage, *S. aureus* infection or *S. aureus* transmission, ii.) articles assessing effects of OCT (used alone or within bundle strategies) on the occurrence of SSIs (superficial or deep-seated), and iii.) on the occurrence of ICU-acquired or catheter-related infections (catheter-related bloodstream infections or wound infections at the catheter insertion site).

We excluded reports of single cases, but case series, outbreak reports and cohort studies were included (even if there was no control group). All articles describing only experimental *in vitro* effects (e.g., on bacterial isolates, biofilms) and veterinary subjects were excluded. Articles describing effects when applying OCT for the topical antiseptic treatment of chronic wounds (e.g., ulcers), oral cavities, mucosa or the urinary and genital tract were also excluded, if the outcomes measured were not related to SSIs, ICU-/catheter-related bloodstream infections, catheter insertion site infections, or *S. aureus*-infection, -carriage or -transmission. Reviews were excluded but retrieved and the literature lists of reviews and all included articles were screened for relevant information.

Data extraction

RK assessed which articles met the entry criteria for at least one outcome and performed a data extraction in standardized pre-designed tables (Table 1, Tables S2, S3, S4). The extraction results were then crosschecked by all authors and in case of disagreement, consensus was reached by discussion with the panel of authors. A formal meta-analysis of the results was not performed, as the designs and outcomes of the identified studies were too heterogeneous. Data on the financial support of studies were asssed in Table 1 as mentioned in the respective studies. Data on the type and concentration of the antiseptics used were extracted as mentioned in the respective articles without interpretation (Tables S2-S4). Effect measures were extracted as mentioned in the respective studies (Table S2).

Table S2. Data extraction table.

Study (Ref.)	Outcome(s)	Intervention(s)	Specification of the antiseptics used	Microbiology	Results
**Studies using OCT as a component of bundles to prevent *S. aureus* or MRSA spread and infection among patients with confirmed carriage**					
Allport [19]	Primary outcome: decolonisation efficacy (MSSA on the day of surgery).<br>Secondary outcome: rate of MSSA prosthetic joint infection (PJI).	3,200 nasal MSSA carriers were treated 5d before and 5d after elective hip and knee joint arthroplasty with three different nasal ointments in three phases (group 1: 2010-2017, group 2: 2017-2018, group 3: 2010-2017). Patients in all groups used OCT body wash.	Group 1: nasal mupirocin 2% (Bactroban®, GlaxoSmithKline), 2x/d for 5d before and 5d after surgery.<br><br>Group 2: nasal OCT 0.1% (Octenisan® Nasal Gel, Schülke & Mayr), 2x/d for 5d before and 5d after surgery.<br><br>Group 3: nasal neomycin 0.5% / CHX 0.1% (Naseptin®, Alliance Pharmaceuticals), 4x/d, for 5d before and 5d after surgery.<br><br>All groups: OCT body wash (Octenisan®, Schülke & Mayr), 1x/d for 5d prior to surgery.	Nasal and groin swabs prior to surgery and on the day of surgery. Analysis on colorimetric media to selectively detect MSSA without pre-enrichment.	Groups 1-3 contained 698, 1221 and 1210 patients, respectively.<br>Of those patients where nasal control swabs were performed, 35/322 (10.9%) of all MSSA carriers treated in group 1, 415/830 (50%) of those treated in group 2 and 57/624 (9.1%) of those treated in group 3 still carried MSSA ($p<0.0001$).<br><br>There was no difference in MSSA PJI rates (2/698 in group 1, 3/1221 in group 2, and 2/1210 in group 3, $p=0.452$) or overall PJI rates between the three groups (6/698 in group 1, 3/1221 in group 2, and 8/1210 in group 3, $p=0.108$).

Study (Ref.)	Outcome(s)	Intervention(s)	Specification of the antiseptics used	Microbiology	Results
Aung [20]	Rate of MRSA acquisition on a dermatology ward.	MRSA acquisition was assessed by screening at admission and discharge and compared between a baseline phase and an intervention phase. During the intervention phase, all patients received daily OCT baths. MRSA carriers additionally received intranasal OCT.	OCT baths (unspecified product) 1x/d for 5d.<br><br>OCT nasal gel (Octenisan® MD nasal gel, Schülke & Mayr), unspecified how often daily for 5d after admission.	All patients were screened for MRSA on admission and those who stayed for >24 hours again at discharge. Swabs from nares, axillae, and groin were cultured on selective chromogenic agar.	802 patients were included (400 in the baseline and 402 in the intervention periods). MRSA carriage rate at admission was 15.9% vs. 11.8% during baseline and intervention period.<br>Among MRSA carriers, 6 of 62 (9.7%) vs. 10 of 47 (21.3%) screened negative for MRSA at discharge. Among non-MRSA carriers, the rates of MRSA-acquisition on the ward were 14/329 (4.3%) vs. 8/351 (2.3%). Patients admitted during the intervention period were 63% less likely than those admitted during the baseline period to acquire MRSA during their stay (adjusted OR (aOR) 0.37; 95% CI, 0.14–0.98; p=0.046). The length of stay on the ward was independently associated with MRSA-acquisition (aOR, 1.12; 95% CI, 1.05–1.18; p<0.001).

**Study (Ref.)**	**Outcome(s)**	**Intervention(s)**	**Specification of the antiseptics used**	**Microbiology**	**Results**
Buehlmann [21]	Efficacy of MRSA decontamination	Decolonization treatment for MRSA carriers including mupirocin nasal ointment, CHX mouth rinse, full-body wash with chlorhexidine soap for 5d. Intestinal and urinary-tract colonization were treated with oral vancomycin and cotrimoxazole, vaginal colonization with povidone-iodine or CHX ovula or OCT solution. Other antibiotics were added, if this treatment failed.	Mupirocin nasal ointment (Bactroban®; GlaxoSmithKline), 2x/d for 5d, oral rinsing with 0.2% CHX solution (Dentohexin®; Streuli Pharma) 3x/d body washing with 4% CHX soap (Lifoscrub®; B. Braun), 1x/d.<br><br>Vaginal colonization was treated with povidone-iodine ovula (Betadine®; Mundipharma Medical Company) 1x/d for 5d or hexetidine ovula (Vagi-Hex®; Drossapharm) 2x/d for 6d or (Octenisept®; Schülke & Mayr) 2x/d for 6d.	Swabs from nose, throat, inguinal area, perianal area, rectum, vagina, wounds, insertion sites, or urine [for patients with urinary-tract colonization] obtained 2-3d after treatment every 2-3 d, then at each new admission. Culture technique unspecified.	62 MRSA carriers were included. 65% of patients required oral antibiotic treatment. Decolonization was successful in 54/62 patients in an intent-to-treat analysis and in 51/52 patients in an on-treatment analysis.

Study (Ref.)	Outcome(s)	Intervention(s)	Specification of the antiseptics used	Microbiology	Results
Chow [22]	MRSA acquisition rate per 1,000 patient-days	Universal daily CHX whole body bathing throughout the whole period (2013-2019), intranasal OCT for MRSA colonizers added from September 2017 onwards (i.e. the intervention).	Body wash with CHX gluconate 4% (Microshield®; Johnson & Johnson), unspecified how often daily for 5d.<br><br>Nasal OCT (Octenisan® md nasal gel; Schülke & Mayr), unspecified how often daily for 5d.	Screening on admission and at discharge. Swabs from the nares, axillae, and groin cultured on selective chromogenic MRSA agar media. In addition, clinical samples as clinically indicated.	Mean monthly MRSA acquisition rate was 7.0/ 1000 patient-days (pd) pre intervention vs. 4.4/1000 pd post intervention ($p<0.001$).<br>After adjustment for MRSA carriage at admission and hand hygiene compliance, a constant trend was observed before adding nasal OCT (adjusted mean coefficient: 0.012; 95% CI -0.037 to 0.06), with an immediate decrease when OCT was added (-2.145; -0.248 to -0.002; p=0.033), followed by a significant reduction in MRSA acquisition post implementation (-0.125; -0.248 to -0.002; p=0.047).

Study (Ref.)	Outcome(s)	Intervention(s)	Specification of the antiseptics used	Microbiology	Results
Chow [23]	Prevalence of MRSA colonization determined in three point prevalence screenings (June/July 2014, 2015, 2016)	Hospital A: Universal daily chlorhexidine bathing for all patients throughout the whole period. Nasal OCT for 5d for known MRSA carriers in 03-06/2016.<br><br>Hospital B: Universal daily OCT bathing and nasal OCT for 5d for known MRSA carriers from 03-07/2016.<br><br>Hospital C: No antiseptic bathing and no nasal OCT for MRSA carriers or other patients.	Bathing with chlorhexidine gluconate 4% (Microshield®, Johnson & Johnson), 1x/d unspecified how long ("universal").<br><br>Bathing with OCT (Octenisan® wash lotion, Schülke & Mayr), 1x/d unspecified how long ("universal").<br><br>Nasal OCT (Octenisan® md nasal gel, Schülke & Mayr), unspecified how often daily for 5d.	Swabs from the nares, axillae and groin were taken in point prevalence periods.<br>Swabs cultured on selective chromogenic MRSA agar media without pre-enrichment.	MRSA prevalence was similar among the hospitals in 2014 (Hospital A: 33.0%, Hospital B: 26.6%, Hospital C: 32.2%, p=0.453) and 2015 (Hospital A: 38.5%, Hospital B: 48.1%, Hospital C: 43.4%, p=0.288). It decreased from 2015 to 2016 in Hospital A (2015: 38.5%, 2016: 19.3%, p=0.007) and Hospital B (2015: 48.1%, 2016: 34.4%, p=0.001), but remained stable in Hospital C (2015: 43.4%, 2016: 38.9%, p=0.554).

**Study (Ref.)**	**Outcome(s)**	**Intervention(s)**	**Specification of the antiseptics used**	**Microbiology**	**Results**
Danilevicius [24]	Efficacy of MRSA decontamination	MRSA carriers were treated with several OCT-based products in parallel (prolonged application if controls after 7d were still positive).	For 7 days: Octenidol® gargling (3x/d), whole body wash Octenisan® (1x/d) or whole body wash with wipes Octenisept® (1x/d), nasal OCT 0.05% (3x/d), wound Octenilin® solution (1x/d). If this treatment failed, it was repeated for another 7d.	Swabs from nostrils, perineum, wounds if present (culture technique unspecified) taken 2d after end of treatment.	Decontamination was achieved in 24/36 patients (67%) after 7-14d.
Hansen [25]	Efficacy of MRSA decontamination	MRSA carriers were treated with OCT body wash and mupirocin nasal ointment (only if nasal carriage was detected). The authors state that both the measures and the swabs "should have been" performed or taken.	1:1 diluted OCT (unspecified product, Schülke & Mayr) 1x/d (unspecified for how many days), mupirocin ointment 3x/d for 5d (GlaxoSmithKline) only if nasal colonization was found.	Swabs from nose, hairline, perineum, wounds and other body sites (if considered necessary) taken 3x on consecutive days starting >3d after end of decolonization therapy and systemic antistaphylococcal antibiotics. Culture technique unspecified.	It was not assessed whether the planned interventions were performed. 5/87 patients (6%) decolonized from MRSA carriage at discharge (based on definition that three 3 consecutive negative series of swabs were taken), 7/87 (8%) had two series and 6/87 (7%) one series of swabs without MRSA before discharge. In 52/87 (60%) decolonization failed and for 17/87 data were missing.

Study (Ref.)	Outcome(s)	Intervention(s)	Specification of the antiseptics used	Microbiology	Results
Harris [26]	Primary outcome: number of patients newly acquiring MRSA.<br><br>Secondary outcome: rate of clinical MRSA infections in patients identified as colonized at the time of entry to the ward.	Before results of active admission screenings were available, all patients on ICUs and general wards received OCT body washes (mostly 48h). Then, patients identified as MRSA carriers received daily OCT body washes (i.e. intervention) on defined wards vs. body washes with soap and water on other wards. After 6 months and a wash-out period of 1 month, the intervention- and control-wards were switched. MRSA patients were placed in contact precautions.	OCT lotion or sponging with OCT mitts (Octenisan®, Schülke & Mayr), 1x/d with 1 min. contact time. Performed by all patients with pending screening results and thereafter by MRSA positive patients in intervention group (for at least 7d, and repeatedly, if still positive thereafter).	Swabs from the anterior nares, groin and any wounds cultured on MRSA selective media without pre-enrichment.<br>MRSA screening was performed at admission and weekly thereafter.	10935 patients included (5277 in intervention group and 5658 in control group). New MRSA acquisitions (nosocomial) affected 187 (3.3%) in the control group vs. 156 (3.0%) in the intervention group (OR 0.89; 95% CI 0.72–1.11; p=0.31). No significant differences in MRSA clinical infections (36/5277 vs. 40/5658, OR 0.99; 95% CI .63–1.55; p=0.96) or MRSA bacteraemia events (7/5,277 vs. 6/5658, OR 1.25; 95% CI 0.42–3.73; p=0.69) between the intervention and control group.

Study (Ref.)	Outcome(s)	Intervention(s)	Specification of the antiseptics used	Microbiology	Results
Kaminski [27]	Efficacy of MRSA decontamination	MRSA screening of healthcare workers in a trauma centre. If permanent carriage was found, a decolonization protocol was established (and partly household members screened).	Decolonization series: Mupirocin nasal ointment 3x/d for 5d (Turixin®, SmithKline Beecham).<br><br>Throat rinse with OCT solution (Octenisept®, Schülke & Mayr) diluted 1:4) 1x/d for 5d.<br><br>Body wash with OCT (Octenisept®, Schülke & Mayr, diluted 1:1) 1x/d for 5d.	Swabs from nose, throat and perineum cultured on 4% NaCl blood agar after enrichment in 10% NaCl broth.	17/324 healthcare workers carried MRSA, of which 11 were permanent carriers. 9/11 permanent carriers were decolonized after an average of 1.5 decolonization series. 2/11 received additional systemic antibiotics. Follow-up of the former carriers for 1 year.

Study (Ref.)	Outcome(s)	Intervention(s)	Specification of the antiseptics used	Microbiology	Results
Pichler [28]	Efficacy of MRSA decontamination	MRSA carriers identified in screening were isolated and received OCT treatment.	OCT-based body and hair washing 1x/d (Octenisan® wash mitts and Octenisan® wash caps); mouth rinse 3x/d (Octenidol® md); nasal-gel 3x/d (Octenisan® md nasal gel). OCT antiseptic (Octenisept®) was used 1x/d to disinfect tracheostomata, catheter exit sites and decontaminate dental prostheses (all products Schülke & Mayr and applied for 5d). If, MRSA was still detected after a 5d-course, the procedure was repeated.	Screening at admission. Swabs of nares, axillae, inguinal skin, wounds, tracheostomata, percutaneous endoscopic gastrostomy stomata (PEG stomata) and any catheter exit sites cultured on MRSA selective media without pre-enrichment.	After 5d, 17/25 patients remained MRSA positive. This declined to 14/25 after a second decontamination therapy and 8/25 after a third decontamination therapy. No patients developed an MRSA-related infection or an allergic reaction to OCT-based products.

Study (Ref.)	Outcome(s)	Intervention(s)	Specification of the antiseptics used	Microbiology	Results
Rengelshausen [29]	Efficacy of MRSA decontamination	MRSA carriers were treated with OCT washes and mouth irrigation, and mupirocin nasal ointment. Treatment was continued until hospital discharge or successful eradication.	0.1% OCT plus 2% phenoxyethanol (Octenisept®) body wash 1x/d, and mouth irrigation 2x/d. mupirocin (2%) nasal ointment 3x/d for 5d.	Swabs taken daily from hairline, nostrils, oral cavity, sputum, axilla, genital and anal skin, and wounds (culture technique unspecified) until 5d of negative cultures from all sites.	4/5 patients included stayed colonized after a mean of 25d.

Study (Ref.)	Outcome(s)	Intervention(s)	Specification of the antiseptics used	Microbiology	Results
Richter [30]	Efficacy of *S. aureus* decontamination	In an outbreak situation, *S. aureus* carriers (mostly MSSA) among *staff members of a neonatal ICU* decolonized themselves at home with OCT-based products. A second decontamination was performed, if the first one failed (same procedure). For a third trial, mupirocin nasal ointment was used instead of OCT-based nasal gel, combined with professional dental cleaning and tests/treatments of household members	OCT-based nasal gel, mouth rinse, washing solution (products and application unspecified) for 5d.<br><br>Mupirocin nasal ointment (product and application unspecified) for 5d.	Swabs from the nose and pharynx (culture technique unspecified) taken 3d after end of treatment on three consecutive days.	3/40 treated carriers of *S. aureus* were tested negative after the first trial. A second trial was performed in 11 carriers, of which 8 stayed positive. After a third trial all of these 8 carriers were *S. aureus* negative.

Study (Ref.)	Outcome(s)	Intervention(s)	Specification of the antiseptics used	Microbiology	Results
Rohr [31]	Efficacy of MRSA decontamination	MRSA carriers (only with at least one non-nasal carriage site) were decolonized with intranasal mupirocin and OCT body wash.	Mupirocin (Turixin®, SmithKlineBeecham) 3x/d for 5-7d.<br><br>Whole-body wash with OCT (Octenisept®, Schülke & Mayr), 1x/d for 5d.	Swabs from nose, axilla, groin (cultured on sheep blood agar with 4% NaCl after NaCl enrichment). Neck and forehead sampled with contact agar plates (24 cm^2).	Of 32 patients included for decolonization, MRSA was decolonized at all sites in 53% after 24–48 h, and in 64% 7-9d after the end of the treatment. Decolonization was most successful at the nasal site (88.5% reduction of carriage).
Sloot [32]	Efficacy of MRSA decontamination	Known MRSA carriers were treated with nasal mupirocin and OCT whole body wash	Whole-body washes including the hair 1x/d for 5d with 1000 ml Octenisept® (0.1 % OCT, 2% phenoxyethanol, Schülke & Mayr) diluted 1:1 with tap water.<br>Nose was treated 3x/d for 5d with mupirocin nasal ointment (Turixin®, SmithKline Beecham Pharma).	Swabs taken at admission, then on day 4 of the therapy and days 1, 4, and 7 thereafter. Swabs from nares, hairline, pharynx, groin, axilla, wounds, sub-mammary region. Cultured directly on non-selective blood agar.	MRSA decolonization was achieved in 21 of 28 (75%) cases

Study (Ref.)	Outcome(s)	Intervention(s)	Specification of the antiseptics used	Microbiology	Results
Spencer [33]	MRSA acquisition. Secondary outcomes: ICU-acquired MRSA bacteraemia and all ICU-acquired bacteraemia cases.	Pre-intervention phase: MRSA non-colonized patients washed 1x/d in soap and water. Intervention phase: MRSA non-colonized patients washed with OCT for 5d, then washing with soap and water on day 6 and 7 followed by surveillance swabbing on day 7.	OCT wash 1x/d for 5d (Octenisan®, Schülke & Mayr); contact time 3 minutes, with hair washing on days 2 and 4).<br><br>MRSA carriers: additionally 3x/d nasal 2% mupirocin (Bactroban®, GlaxoSmithKline).	Swabs (nares, perineum) at admission and weekly (day 7, 14 etc.) thereafter, cultured on chromogenic MRSA selective media without pre-enrichment.	Total numbers of MRSA acquisitions reduced from 25/1206 patients admitted to 6/1225 patients admitted. In Poisson regression, mean number of cases per month post intervention was 76% lower (95% CI: 42-90%; $p < 0.01$).<br>ICU-acquired bacteraemia not significantly changed (n=37 pre-intervention vs. n=31 post intervention, p not shown). ICU-acquired MRSA bacteraemia n=3 pre-intervention vs. n=0 post intervention (p not shown).

Study (Ref.)	Outcome(s)	Intervention(s)	Specification of the antiseptics used	Microbiology	Results
Wisgrill [34]	Incidence rate of MSSA attributable infections	Very low birth weight infants (VLBWI) were screened for *S. aureus* carriage. MSSA-colonized VLBWI with central and/or peripheral lines were decolonized for 5d. After control, the protocol was repeated, if a central and/or peripheral line was still used. This intervention was compared to a pre-interventional phase without screening and decolonization.	Nasal mupirocin gel 3x/d for 5d (unspecified product).<br><br>skin washing with 0.1% OCT solution 1x/d for 5d (unspecified product); repeated in some cases.	Swabs once weekly from the nares and skin (cultured on *S. aureus* selective chromogenic media).	552 VLBWI were included in the pre-intervention period and 504 in the post-intervention period. The incidence rate of MSSA-attributable infections was 1.63 [95% CI 1.12–2.31] vs. 0.83 [95% CI 0.47–1.35] in the pre- and post-intervention period (p = 0.024)
**Studies using OCT for pre-incisional antisepsis or universally among defined patients and assessing effects on surgical site infections**					
Hachenberg [35]	Complications among female patients undergoing implant-base breast reconstruction	OCT wash lotion for 5d before breast reconstructive surgery in intervention group vs. no pre-operative treatment in control group. Antisepsis of the incision site undefined.	Octenisan® wash lotion (Schülke & Mayr), 1x/d for 5d.	No microbiological cultures taken.	Compliance with preoperative washing was 48.5% (96 patients). Minor complications in 37/101 in control vs. 33/96 patients in intervention group (p=0.74) including 8/101 vs. 10/96 infections (p=0.543). Major complications (with implant loss) in 7/101 in control vs. 5/96 in intervention group (p=0.61) including infections (2/101 vs. 3/96, p=0.584).

Study (Ref.)	Outcome(s)	Intervention(s)	Specification of the antiseptics used	Microbiology	Results
Jeans [36]	Rate of prosthetic joint infections (PJI) for total hip and total knee replacements	Pre-operative screening for MSSA carriage introduced in 2010 among patients undergoing elective arthroplasty. All patients received OCT body wash 5d before surgery, and all MSSA carriers additionally received nasal mupirocin for 5d before and after surgery, respectively	Body washes with Octenisan® (Schülke & Mayr), 1x/d for 5d prior to surgery.<br><br>Mupirocin (Bactroban®, Glaxo- SmithKline), 4x/d for 5d before and 5d after surgery.	Swabs from nose and groin taken pre-operatively and cultured on chromogenic *S. aureus* selective agar.	Overall PJI rate fell from 69/3593 (1.9%) to 131/9318 (1.4%) (p = 0.03). The rate of PJI due to MSSA fell from 28/3593 (0.75%) to 23/9318 (0.25) (p< 0.0001). The effect was pronounced for overall infections after total hip replacements (48/1624 (3.0%) vs. 64/4293 (1.5%); p=0.0002), as well as MSSA infections after total hip replacements (19/1624 (1.2%) vs. 10/4293 (0.2%), p<0.0001).

Study (Ref.)	Outcome(s)	Intervention(s)	Specification of the antiseptics used	Microbiology	Results
Karl [37]	Surgical site infections (SSI) among patients undergoing arterial reconstruction surgery	Intervention phase with newly introduced bundles consisting of: pre-operative antiseptic nasal gel, body wash, hair clipping instead of shaving, pre-incisional antiseptics changed from alcohol to alcohol/OCT, optimized timing for antibiotic prophylaxis, optimized normothermia management, optimized surgical and wound care techniques.	OCT for pre-incisional antiseptics (unspecified product).<br><br>Nasal gel and whole body wash: unspecified whether OCT-based or not.	Not specified.	Comparing patients in the pre-intervention phase (n=195) to patients in the intervention phase (n=233), there was a reduction of SSIs from 22 to 14 (11.3% (95 %-CI: 7.2-16.6%) vs. 5.5% (95%-CI: 2.7-9.9%).

Study (Ref.)	Outcome(s)	Intervention(s)	Specification of the antiseptics used	Microbiology	Results
Kohler [38]	Surgical site infections (SSI) among patients undergoing cardiac surgery.	Nasal mupirocin plus body washes / showering with chlorhexidine digluconate 4% or, for bedridden patients, OCT impregnated washing gloves were started at hospital admission and continued after cardiac surgery for a minimum of 5d (i.e. intervention group). SSI rates were determined and compared with the period before the intervention (control group).	All patients: mupirocin nasal ointment (Bactroban®, GlaxoSmithKline, Brentford, UK), 2x/d for 5d (at least).<br><br>For non-bedridden patients: CHX digluconate 4% washing solution (Lifo-Scrub; B. Braun Medical), 1x/d for 5d (at least)<br><br>For bedridden patients: OCT impregnated washing gloves (Octenisan® wash mitts; Schülke & Mayr), 1x/d for 5d (at least).	Cultures taken when clinically indicates in case of SSI.	Overall SSI rate 8.6% (81/945) in control phase vs. 6.9% (58/842) in intervention phase ($p=0.19$). In multivariable analysis, the intervention was associated with an odds ratio of 0.61 (95% confidence interval, 0.41-0.91; $p=0.015$) regarding any SSI. This effect was only due to a decrease in superficial SSI (10/646 vs. 8/196, $p=0.032$), and not observed for deep-seated infections (27/646 vs. 13/196, $p=0.16$). The risk reduction was hence 0.29 (0.15-0.58, $p<0.001$) for superficial SSI and 1.06 (0.65-1.74, $p=0.81$) for deep-seated SSI.<br>Effects of OCT vs. CHX in the intervention group were not stratified. It remained unspecified how many patients were treated with OCT and how many with CHX.

**Study (Ref.)**	**Outcome(s)**	**Intervention(s)**	**Specification of the antiseptics used**	**Microbiology**	**Results**
Matiasek [39]	Incidence of wound dehiscence, hematoma; and infection among patients receiving blepharoplasties	Disinfection of the eye and face with OCT prior to blepharoplasty thrice in supine position (face scrubbed from medial to lateral and hairline to the claviculae including the anterior ear). The face was then draped with sterile cloth. After the third wash, OCT was left on to dry.	Octenisept® (Schülke & Mayr) for skin preparation before surgery.	No microbiological cultures taken.	0/352 patients with blepharoplasty had a wound infection, 6.3% developed wound dehiscence, 7.4% hematoma.
Reiser [40]	Surgical site infections (SSI) among patients undergoing cardiac surgery	Nasal application of OCT ointment, beginning on the day before surgery, and showering the night before and on the day of surgery with OCT soap.	Nasal OCT (Octenisan® nasal gel, Schülke & Mayr), 3x /d (unspecified how many days). Shower the night before and on the day of surgery with OCT liquid soap (Octenisan® wash lotion, Schülke & Mayr) incl. hair, rinse with water after a pause of at least 1 min.<br><br>An alcohol-based povidone-iodine solution (Braunoderm®, B. Braun) was used for antiseptics at the incision sites.	Cultures taken when clinically indicates in case of SSI.	Overall, no difference in SSI rates between the control and intervention group (15.4% vs 13.3%, p=0.39). Rate of harvest site SSIs (2.5% vs 0.5%, p=0.01) and, in patients with median sternotomy significantly lower rate of organ/space sternal SSIs (1.9% vs 0.3%, p=0.04) in intervention group, but there was a trend towards more deep incisional sternal SSIs in the intervention group (1.2% vs 2.9%, p=0.08). In multi-variate analysis no significant protective effect of the intervention was found (odds ratio 0.79, 95% confidence interval 0.53-1.15, p=0.27).

**Study (Ref.)**	**Outcome(s)**	**Intervention(s)**	**Specification of the antiseptics used**	**Microbiology**	**Results**
Studies using OCT universally among defined patients and assessing effects on ICU-/catheter-related bloodstream infections or insertion site infections					
Baier [41]	incidence rate of central-line associated bloodstream infection (CLABSI)	All patients at a burn ICU washed daily with OCT and received OCT gel intranasally.	OCT washing gloves (unspecified product), 1x/d for 6d a week.<br><br>OCT nasal gel (unspecified product) 1x/d.<br><br>OCT mouth rise (unspecified product) at least 1x/d.<br><br>This procedure was performed as long as the patient stayed on the ward.	Unspecified. Admission screening for multidrug-resistant bacteria was performed from nasopharyngeal and rectal swabs and respiratory Secretions (technique unspecified). A weekly screening for multidrug-resistant bacteria was established, if there was one patient on the ward (technique unspecified).	CLABSI incidence decreased from 2.03/1000 central venous catheter days in baseline phase to 0.82/1000 central venous catheter days in the decolonization phase; incidence rate ratio was 0.40 (95% CI: 0.06–1.71, p=0.254). Incidence of CLABSI per 100 patients with CVC was 1.91 in the pre-phase and 1.07 after the intervention, IR = 0.56, (0.12–2.61), p=0.491. Incidence rate of nosocomial MRSA cases per 1000 patient days was 0.52 vs. 0 (p=0.505) and of other nosocomial multidrug-resistant bacteria clusters per 1000 patient days was 0.69 vs. 0 (p=0.320) before vs. after the intervention, respectively.

Study (Ref.)	Outcome(s)	Intervention(s)	Specification of the antiseptics used	Microbiology	Results
Bilir [42]	Primary outcome was Catheter-related sepsis (unspecified criteria). Secondary outcome was colonization of the insertion site.	Patients with intravascular catheters randomized into three groups to receive antiseptic treatment (chlorhexidine, povidone iodine, OCT) when inserting the catheter as well as for daily insertion site care thereafter.	Group 1: 4% CHX<br>Group 2: 10% povidone iodine<br>Group 3: OCT (unspecified concentration).<br><br>All groups: used for insertion and site care (unspecified how often and which product).	Cultures taken from the catheter insertion site daily and at the catheter hub (technique of culture retrieval and processing in the laboratory unexplained).	Groups 1-3 contained 19 patient each (57 patients in total).<br>Catheter-related sepsis occurred among 0% of patients in group 1, 10.5% in group 2 and 20.5% in group 3 ($p<0.001$). Catheter insertion site colonization was found in 0% in group 1, 26.3% in group 2 and 21.5% in group 3 ($p<0.001$).<br>(Absolute numbers of infections not provided. Definition of infection remains unclear.)
Denkel [43]	Incidence density of CLABSI per 1,000 central-line (CL) days	Wards randomly assigned to one of two decolonization regimes (interventions) or routine care (control).<br><br>Intervention included universal daily bathing with CHX or OCT, whereas the control group used water and soap (routine care group).	CHX group: 2% chlorhexidine-impregnated cloths (Sage® 2% Chlorhexidine Gluconate Cloths) below the jawline and none-CHX-containing disposable cloths above the jawline.<br><br>OCT group: 0.08% OCT disposable wash mitts (Octenisan®, Schülke).	Blood cultures taken as clinically indicated.	22897 patients in the CHX group, 25127 in the OCT group, and 28791 in the routine care group were included. Incidence densities were 0.90 CLABSI per 1000 CL days (95% CI 0.67-1.19) in the CHX group, 1.47 (95% CI 1.17-1.81) in the OCT group, and 1.17 (95% CI 0.93-1.45) in the routine care group. Adjusted incidence rate ratios of CLABSI (compared with routine care) were 0.69 (95% CI 0.37-1.22, p=0.28) in the CHX group and 1.22 (95% CI 0.54-2.75, p=0.65) in the OCT group.

Study (Ref.)	Outcome(s)	Intervention(s)	Specification of the antiseptics used	Microbiology	Results
Dettenkofer [44]	1. Skin colonization at the insertion site.<br><br>2. Quantitative culture from catheter tips.<br><br>3. Catheter-associated bloodstream infections	Patients with non-tunnelled central venous catheters were randomized to receive antiseptic treatment with OCT/alcohol vs. alcohol-based disinfection of the catheter insertion site.	Insertion site treatment with 0.1% OCT with 30% 1-propanol and 45% 2-propanol (in the OCT group) and 74% ethanol with 10% 2-propanol (in the control group). The antiseptics were delivered in bottles that were indistinguishable for the applicant (manufacturer not mentioned).	Quantitative culture from insertion site before insertion and every 3 +/I 1 d, as well as from catheter tips with defined sampling and culture techniques. Blood cultures as clinically indicated.	Quantitative skin cultures from the insertion site showed a lower number of colony forming units (CFU) for 187 patients in the OCT group vs. 178 in the control group (adjusted mean of 21 vs. 100 CFU, p<0.0001). Quantitative catheter tip cultures showed lower proportion of patients with tips ≥ 15 CFU/5 cm for 165 patients in the OCT group vs. 157 in the control group (13 vs. 28 patients, p=0.009). The occurrence of catheter-associated bloodstream infections was non-significantly lower for 194 patients in the OCT group vs. 193 in the control group (8 vs. 16 patients, p=0.081).

Furtwängler [45]	Number and incidence of patients with bloodstream infection (BSI)	Two techniques for central venous access device (CVAD) care among paediatric cancer patients.<br><br>Period 1 (P1, 2009-2011): Insertion cite care: Octenisept®, PVP-iodine or polyhexanide if local inflammation. Hub with Softasept® N. Flushing with NaCl 0.9% prepared manually. Minimal flushing frequency 2x/week.<br><br>Period 2 (P2, 2011-2013): Insertion cite care: Octenisept®, medical honey if local inflammation. Hub with Octeniderm®. Flushing with commercial NaCl 0.9% (BD Posiflush®, Becton Dickinson). Minimal flushing frequency 1x/week.	Softasept® N (in 100 mL: ethanol (100%) 74.1g, 2-propanol 10g), B. Braun).<br><br>Octenisept® (in 100 mL: OCT 0.1g, 2g phenoxyethanol, Schülke & Mayr).<br><br>Octeniderm® (0.1 g OCT, 30.0 g 1-propanol and 45.0 g 2-propanol in 100 g solution, Schülke & Mayr).	Two blood cultures (aerobic and anaerobic) were collected from patients with fever after disinfection of the CVAD hub before the first dose of intravenous antibiotics.	Median time between implantation and removal of the CVAD 192d in P1 and 191d in P2.<br><br>Non-significant reduction in all outcomes: 28 BSI were diagnosed in 22/84 patients in P1 and 15 BSI in 12/81 patients in P2 (p=0.08). The incidence density of BSI per 1000 patient-days was 0.44 (95% CI 0.29–0.62) for P1 vs. 0.34 (0.19–0.53) for P2 (p=0.2) and the incidence rate of BSI per 1,000 inpatient CVAD utilization days was 7.76 (5.16–10.86) in P1 vs. 4.75 (2.66–7.43) in P2 (p=0.1).

**Study (Ref.)**	**Outcome(s)**	**Intervention(s)**	**Specification of the antiseptics used**	**Microbiology**	**Results**
Gastmeier [46]	Incidence of ICU-acquired BSI and cases of multidrug-resistant organisms (MDRO)	Study performed on 17 ICUs.<br>In baseline period: general admission screening for MRSA and screening of defined risk groups for VRE and Gram-negative MDRO followed by isolation of carriers/infected.<br>In intervention: universal nasal treatment with OCT gel and OCT wash cloths.<br>No active screening except for Gram-negative MDRO risk patients, no isolation.	OCT nasal gel (unspecified product (Schülke & Mayr), unspecified how often daily) for 5d after ICU admission.<br><br>OCT wash cloths 1x/d (unspecified product, Schülke & Mayr) for the whole ICU stay.	Microbiological technique for the admission screenings in the baseline period undefined. Blood cultures taken as clinically indicated. No microbiological screening during the course of the ICU stay carried out in the baseline or intervention phase.	29532 ICU patients (16677 surgical and 12855 medical) included.<br>In baseline period the incidence density of ICU-acquired BSI was 5.1 / 1000 patient days and the ICU-acquired MRSA rate was 0.97 / 1000 patient days.<br>The intervention led to no significant effect on surgical ICUs, but to a reduction of ICU-acquired BSI on medical ICUs (incidence rate ratio 0.78; 95% CI 0.65 – 0.94) and to a reduction of ICU-acquired MRSA on medical ICUs (incidence rate ratio 0.58; 95% CI 0.41 – 0.82).<br>No effect on ICU-acquired VRE and Gram-negative MDRO detected.

Study (Ref.)	Outcome(s)	Intervention(s)	Specification of the antiseptics used	Microbiology	Results
Messler [47]	VRE cases per 100 patients at admission and nosocomial VRE cases per 1,000 patient-days. Incidence of bloodstream infections (BSI) with any pathogen per 1,000 patient-days.	On one ICU, pre-intervention: patients were screened for VRE on admission and twice weekly thereafter. Intervention: OCT-based whole-body wash of all patients	Octenisan® wash cloths (octenidine 0.3%, Schülke & Mayr) 1x/d during the ICU stay. After 2 min, removed with new wash clothes soaked with warm water.	All ICU patients were screened for VRE at admission and twice weekly with a rectal swab cultured on chromogenic VRE media without pre-enrichment. Blood cultures as clinically indicated.	BSI rate 2.98/1000 patients-days before intervention (n=22 cases) vs. 2.06/1000 patient-days post intervention (n=15), p=0.147.<br>VRE admission prevalence and mean incidence density of nosocomial cases were 4/100 patients and 7.55/1000 patient-days before the intervention vs. 2.41/100 patients and 2.61/1000 patient-days post intervention (p<0.001).
Tietz [48]	Primary outcome: Bacterial skin contamination in quantitative skin cultures taken before insertion and repeatedly thereafter. Secondary outcome: Catheter-related bloodstream infections.	For insertion of non-tunnelled central venous catheters (CVC) povidone–iodine was used. Then, OCT was applied for disinfection at the insertion site and during dressing.	Octenisept® (0.1% solution, containing 0.1 g of OCT and 2 g 2-phenoxyethanol per 100 mL, Schülke & Mayr).	Quantitative skin cultures from the CVC insertion site, hubs and catheter tips were taken and cultured with defined techniques before insertion, weekly thereafter and at catheter removal.	57 (13.2%) of 433 quantitative skin cultures from 62 patients showed growth of microorganisms (mostly coagulase-negative staphylococci). The mean colony count in colony-forming units (cfu)/24 cm² decreased over time (no statistical evaluation).<br><br>6 / 62 patients had a documented catheter-related bloodstream infection. The incidence density of catheter infections was 2.39 per 1,000 catheter-days.

Study (Ref.)	Outcome(s)	Intervention(s)	Specification of the antiseptics used	Microbiology	Results
Vogelsang [49]	Catheter infections among patients receiving elective major abdominal surgery with thoracic epidural catheterization	Skin antiseptic for epidural puncture was changed from propan-2-ol (before; 01/2010-06/2014) to propan-2-ol with OCT (after; 07/2014-12/2018).	Before: Propan-2-ol (Cutasept G®, propan-2-ol 63 g in 100 g Paul Hartmann).<br><br>After: Propan-1-ol/propan-2-ol with OCT (Octeniderm®, OCT 0.1 g, propan-1-ol 30 g, propan-2-ol 45 g in 100 g Schülke & Mayr).	Cultures as clinically indicated.	6/1,120 patients with propan-2-ol disinfection vs. 10/1,635 patients with OCT disinfection had a catheter-related infection (p=0.797).

Table S3. Overview of octenidine-containing products used for non-nasal application sites.

Study (Ref.)	Application type	concentration	Brand name	Applied x times	For x days
Allport [19]	body wash	NA	Octenisan	1x/day	5 daya
Aung [20]	body wash	NA	NA	1x/day	5 days
Buehlmann[21]	vaginal	NA	Octenisept	2x	6 days
Chow [23]	body wash	NA	NA	1x/day	NA
Danilevicius [24]	body wash	NA	NA or Octenisept	1x/day	at least 7 days
Hansen [25]	body wash	NA	NA	NA	NA
Harris [26]	body wash	NA	Octenisan	1x/day	at least 7 days
Kaminski [27]	body wash	1:1 diluted	Octenisept	1x/day	5 days
Kaminski [27]	throat	1:4 diluted	Octenisept	1x	5 days
Pichler [28]	body wash	NA	Octenisan	1x/day	5 days
Pichler [28]	mouth, tracheostoma, catheter exit sites, dental prostheses	NA	Octenidol (mouth), Octenisept (other sites)	3x (mouth), 2x (denture), 1x (other sites)	5 days
Rengelshausen [29]	body wash	0.1%	Octenisept	every two days	various
Rengelshausen [29]	mouth	0.1%	Octenisept	2x	various
Richter [30]	body wash	NA	NA	1x/day	5 days
Richter [30]	mouth	NA	NA	3x	5 days
Rohr [31]	body wash	NA	Octenisept	1x/day	5-7 days
Sloot [32]	body wash	0.1%, 1:1 diluted	Octenisept	1x/day	5 days
Spencer [33]	body wash	NA	Octenisan	1x/day	5 days
Wisgrill [34]	body wash	0.1%	NA	1x/day	5 days
Hachenberg [35]	body wash	NA	Octenisan	1x/day	5 days
Jeans [36]	body wash	NA	Octenisan	NA	5 days
Karl [37]	skin preparation	NA	NA	before surgery	before surgery
Kohler [38]	body wash	NA	Octenisan	1x/day	minimum 5d
Matiasek [39]	skin preparation	NA	Octenisept	before surgery	before surgery
Reiser [40]	body wash	NA	Octenisan	1x before surgery	1x before surgery
Baier [41]	body wash	NA	NA	1x/day	as long as on ward
Bilir [42]	catheter insertion and care	NA	NA	every catheter care	every catheter care
Denkel [43]	body wash	0.08%	Octenisan	1x/day	NA
Dettenkofer [44]	catheter insertion and care	0.1%	NA	every catheter care	every catheter care
Furtwängler [45]	central venous access device care, hub use	0.1%	Octenisept (insertion site), Octeniderm (hubs)	central venous access device care, hub use	central venous access device care, hub use
Gastmeier [46]	body wash	NA	NA	1x/day	as long as on ward
Messler [47]	body wash	0.3%	Octenisan	1x/day	as long as on ward
Tietz [48]	catheter insertion and care	0.1%	Octenisept	every catheter care	every catheter care
Vogelsang [49]	epidural catheter insertion	0.1%	Octeniderm	before insertion	before insertion

NA = information not available

Table S4. Overview of studies in which octenidine-containing products were used for nasal application.

Study (Ref.)	concentration	Brand name	Applied x times	For x days
Allport [19]	0.1%	Octenisan	2x/day	at least 5 days
Aung [20]	NA	Octenisan	NA	5 days
Chow [22]	NA	Octenisan	NA	5 days
Chow [23]	NA	Octenisan	NA	5 days
Danilevicius [24]	0.05%	NA	3x/day	7 days
Pichler [28]	NA	Octenisan	3x/day	at least 5 days
Richter [30]	NA	NA	NA	5 days
Reiser [40]	NA	Octenisan	3x/day	NA
Baier [41]	NA	NA	1x/day	as long as on ward
Gastmeier [46]	NA	NA	NA	5 days

NA = information not available
